# Constipation of Children

**Published:** 1879-08

**Authors:** 


### CONSTIPATION OF CHILDREN.

All the ordinary ills of childhood combined do not work half the misehief that is caused by habitual constipation, from which fully half the children in this country suffer more or less.

Too little attention is paid to this condition of the system by the parents, and the child grows up a perpetual invalid, fated to die early of some one of the score of painful diseases that invariably have their origin in this condition of the system. Now, there is no disease more manageable than this. It only requires the proper remedy, and perseverance in its use until a healthy condition is established. I used to prescribe *Nelaton's Suppositories*, to be divided into small pieces—depending on the age of the child—and used according to the printed directions; but I find there are "*Nelaton's Suppositories for Children*," made from the purest butter of cacao, and mildly medicated to render them safe in all cases. They are sold at fifty cents a box. They are worth fifty dollars a box to any family in which there are children.

[The various medicines referred to in the above article are sold by Hall & Ruckel, wholesale druggists, 218 Greenwich Street, New York. They send them by mail, free, on receipt of price.—Ed.]

